# Supplementary material for: Acetylcholine acts on songbird premotor circuitry to invigorate vocal output
Source: eLife. 2020 May 19;9:e53288. doi: 10.7554/eLife.53288 (PMC7237207; doi:10.7554/eLife.53288)
Supplement: Figure 1—source data 1. [file elife-53288-fig1-data1.docx]

| **Figure panel** | **Feature** | **Carb coeff.** | **Saline coeff.** | **Carb+Atrp coeff.** | **Carb+MEC+MLA coeff.** | **Carb vs. Saline** | **Carb+Atrp vs. Carb+MEC+MLA** |
| --- | --- | --- | --- | --- | --- | --- | --- |
| 1D | Pitch | 0.013 ± 0.0013 | 0.0032 ± 0.0014 | 0.0026 ± 0.0017 | 0.013 ± 0.0016 | p < 1e-3;  n = 103 | p < 1e-3;  n = 57 |
| 1E | Pitch c.v. | -0.12 ± 0.016 | -0.0039 ± 0.018 | -0.039 ± 0.032 | -0.14 ± 0.031 | p < 1e-3;  n = 103 | p = 0.0020;  n = 57 |
| 1G | Tempo | -0.027 ± 0.0036 | -0.0041 ± 0.0040 | -0.0041 ± 0.0035 | -0.022 ± 0.0033 | p < 1e-3;  n = 61 | p = 0.0020;  n = 30 |
| 1I | Amplitude | 0.086 ± 0.029 | 0.019 ± 0.029 | -0.034 ± 0.037 | 0.070 ± 0.037 | p = 0.0060;  n = 127 | p < 1e-3;  n = 69 |

**Figure 1⎯source data 1. Linear mixed effects model analysis of the behavioral effects of carbachol**. For each behavioral feature, we modelled the data (i.e., the normalized values for that behavioral feature) as the sum of a fixed effect of the drug condition and a random effect grouped by bird identity. Models for Carb/Saline and Carb+Atrp/Carb+MEC+MLA were fit separately. Fixed effect coefficients for each drug condition are reported as the coefficient estimate ± standard error. Note the high degree of similarity between the estimated fixed effect coefficients and the reported effect magnitudes. Statistical significance was assessed by a two-sided permutation test, using the difference in fixed effect coefficient estimates as the test statistic. To estimate the null distribution of this test statistic, we randomly reassigned the drug conditions within each bird and fit a new mixed effects model, repeating this process 1000 times.
