# Supplementary material for: Acetylcholine acts on songbird premotor circuitry to invigorate vocal output
Source: eLife. 2020 May 19;9:e53288. doi: 10.7554/eLife.53288 (PMC7237207; doi:10.7554/eLife.53288)
Supplement: Figure 3—source data 1. [file elife-53288-fig3-data1.docx]

| **Figure panel** | **Feature** | **Carb coeff.** | **No LMAN coeff.** | **Carb + No LMAN coeff.** | **No LMAN vs. Carb + No LMAN** |
| --- | --- | --- | --- | --- | --- |
| 3C | Pitch | 0.016 ± 0.0025 | 0.0049 ± 0.0026 | 0.020 ± 0.0026 | p = 1e-3;  n = 70 |
| 3D | Pitch c.v. | -0.13 ± 0.053 | -0.19 ± 0.053 | -0.30 ± 0.053 | p = 0.0060;  n = 70 |
| 3E | Tempo | -0.026 ± 0.0072 | 0.0046 ± 0.0073 | -0.026 ± 0.0072 | p < 1e-3;  n = 49 |
| 3F | Amplitude | 0.12 ± 0.039 | 0.025 ± 0.039 | 0.15 ± 0.038 | p < 1e-3;  n = 88 |

**Figure 3⎯source data 1. Linear mixed effects model analysis of combined carbachol and LMAN inactivation experiments**. For each behavioral feature, we modelled the data (i.e., the normalized values for that behavioral feature) as the sum of a fixed effect of the drug condition and a random effect grouped by bird identity. A single model was fit for all three drug conditions. Statistical significance was assessed by a two-sided permutation test, similar to the analysis described in Figure 1⎯source data 1.
