# Supplementary material for: Acetylcholine acts on songbird premotor circuitry to invigorate vocal output
Source: eLife. 2020 May 19;9:e53288. doi: 10.7554/eLife.53288 (PMC7237207; doi:10.7554/eLife.53288)
Supplement: Figure 4—source data 1. [file elife-53288-fig4-data1.docx]

| **Observed data** | **Carb coeff.**  **(± standard error)** | **Saline coeff.**  **(± standard error)** | **Carb vs. Saline** |
| --- | --- | --- | --- |
| Normalized firing rates (1 per multi-unit site) | 0.099 ± 0.031 | -0.027 ± 0.037 | p = 0.0090;  n = 44 |

**Figure 4⎯source data 1. Linear mixed effects model analysis of multi-unit firing rate changes following microdialysis of carbachol or saline**. We modelled normalized firing rates (drug/baseline) for each multi-unit recording site as the sum of a fixed effect of the drug condition and a random effect grouped by bird identity. For a given multi-unit site, we calculated firing rates aligned to the onset of each syllable (100ms window centered on syllable onset), then averaged these measurements across syllables. Statistical significance was assessed by a two-sided permutation test, similar to the analysis described in Figure 1⎯source data 1.
