# Supplementary material for: Acetylcholine acts on songbird premotor circuitry to invigorate vocal output
Source: eLife. 2020 May 19;9:e53288. doi: 10.7554/eLife.53288 (PMC7237207; doi:10.7554/eLife.53288)
Supplement: Figure 5—source data 1. [file elife-53288-fig5-data1.docx]

| **Observed data** | **Fixed effect estimate**  **(± standard error)** | **Directed vs. Undirected** |
| --- | --- | --- |
| Normalized firing rates (1 per multi-unit site) | 0.089 ± 0.040 | p < 1e-3;  n = 15 |

**Figure 5⎯source data 1. Linear mixed effects model analysis for comparing multi-unit firing rates in directed and undirected song.** We modelled normalized firing rates (directed/undirected) for each multi-unit recording site as the sum of a single fixed effect and a random effect grouped by bird identity. For a given multi-unit site, we calculated firing rates aligned to the onset of each syllable (100ms window centered on syllable onset), then averaged these measurements across syllables. Statistical significance was assessed by a two-sided permutation test, using the fixed effect coefficient estimate as the test statistic. The null distribution of this test statistic was estimated by randomly reassigning the social context condition for each multi-unit site within a given bird, recalculating normalized firing rates, and fitting a new mixed effects model (repeated 1000 times).
