# Supplementary material for: Acetylcholine acts on songbird premotor circuitry to invigorate vocal output
Source: eLife. 2020 May 19;9:e53288. doi: 10.7554/eLife.53288 (PMC7237207; doi:10.7554/eLife.53288)
Supplement: Figure 6—source data 1. [file elife-53288-fig6-data1.docx]

| **Figure panel** | **Feature** | **Pre saline coeff.** | **Atropine coeff.** | **Post saline coeff.** | **Pre saline vs. Atropine** | **Post saline vs. Atropine** |
| --- | --- | --- | --- | --- | --- | --- |
| 6D | Pitch | 0.0060 ± 0.0019 | 0.0032 ± 0.0019 | 0.0061 ± 0.0019 | p = 0.061;  n = 48 | p = 0.062;  n = 48 |
| 6E | Pitch c.v. | -0.41 ± 0.042 | -0.19 ± 0.042 | -0.37 ± 0.042 | p = 0.0030;  n = 48 | p = 0.0020;  n = 48 |
| 6F | Tempo | -0.025 ± 0.0039 | -0.016 ± 0.0041 | -0.020 ± 0.0037 | p = 0.070;  n = 18 | p = 0.098;  n = 18 |

**Figure 6⎯source data 1. Linear mixed effects model analysis of atropine dialysis experiments during directed and undirected song.** For each behavioral feature, we modelled the data (i.e., the normalized values for that behavioral feature) as the sum of a fixed effect of the drug condition and a random effect grouped by bird identity. A single model was fit for all three drug conditions. Fixed effect coefficients for each drug condition are reported as the coefficient estimate ± standard error. Statistical significance was assessed by a one-sided permutation test, similar to the analysis described in Figure 1⎯source data 1.
